# Community-acquired Acinetobacter pneumonia associated with Evans syndrome: a case report

**DOI:** 10.1097/MS9.0000000000004168

**Published:** 2025-10-21

**Authors:** Sana Rasheed, Huda Adnan, Rida Arif, Simran Bai, Amidu Alhassan, Abdul Haseeb

**Affiliations:** a Department of Medicine and Surgery, Jinnah Sindh Medical University, Karachi, Pakistan; b Postgraduate Trainee General Medicine, Jinnah Postgraduate Medical Center, Karachi, Pakistan; c College of Health and Allied Sciences, School of Nursing and Midwifery, University of Cape Coast, Cape Coast, Ghana; d Department of Nursing, All Nations University, Koforidua, Ghana

**Keywords:** Acinetobacter baumannii, autoimmune hemolytic anemia, community-acquired pneumonia, Evans syndrome, immunosuppressive therapy

## Abstract

**Introduction and importance:**

*Acinetobacter baumannii*, a gram-negative bacterium, is a rare cause of community-acquired pneumonia (CAP), especially concerning immunocompromised individuals due to its resistance to multiple antibiotics. It typically presents with fever, productive cough, and shortness of breath, and is associated with high morbidity and mortality. In patients with underlying conditions like Evans syndrome (ES), the management becomes even more challenging. ES is a rare autoimmune disorder characterized by immune thrombocytopenic purpura (ITP) and autoimmune hemolytic anemia (AIHA), which significantly alters the immune response and increases susceptibility to infections.

**Case presentation:**

We present the case of a 19-year-old male with a history of ES who developed severe CAP caused by A*cinetobacter baumannii*. The patient exhibited fever, shortness of breath, and productive cough, with clinical findings of tachycardia, hypotension, pallor, and abnormal laboratory results, including hemolytic anemia and severe thrombocytopenia. Despite initial antibiotic treatment, the infection persisted until polymyxin, effective against multidrug-resistant *A. baumannii*, was introduced. Imaging studies confirmed bilateral lung infiltrates, and the infection exacerbated the patient’s ES, complicating the clinical scenario.

**Clinical discussion:**

This case highlights the increasing role of *A. baumannii* in CAP, particularly in immunocompromised patients, and underscores the importance of timely, targeted treatment. A dual approach involving antibiotics for the infection and corticosteroids for the autoimmune disorder proved crucial for stabilization.

**Conclusion:**

Multidisciplinary care is essential in managing such complex, multifactorial cases. The case emphasizes the need for a collaborative approach involving infectious disease and immunology specialists to improve patient outcomes.

## Introduction

*Acinetobacter baumannii* (AB) is a gram-negative bacterium and a rare cause of community-acquired pneumonia that manifests as shortness of breath, productive cough, and fever^[1]^. The gold standard for diagnosis is gram staining to isolate and identify the gram-negative coccobacilli, a time-consuming procedure that contributes to a high mortality rate^[2]^.

Treatment of opportunistic infections caused by AB is becoming increasingly difficult due to widespread antimicrobial resistance, particularly carbapenems, the last-resort antibiotics for multidrug-resistant *A. baumannii*. Patients with compromised immunity due to comorbidities are at a higher risk of contracting CAP-Ab infection^[3]^. Similarly, Evans Syndrome (ES) is an uncommon medical condition characterized by the co-occurrence of immune thrombocytopenic purpura (ITP) and autoimmune hemolytic anemia (AIHA)^[4]^. Globally, fewer than 4% of patients diagnosed with AIHA or ITP progress to ES^[5]^. It is important to classify ES as either primary/idiopathic or secondary to an underlying disease to guide management and predict prognosis^[6]^.

**HIGHLIGHTS**

- This case reports a rare instance of community-acquired Acinetobacter baumannii pneumonia (CAP-Ab) in a patient with Evans syndrome (ES).
- The patient presented with fever, productive cough, and shortness of breath, alongside severe anemia and thrombocytopenia.
- Sputum culture confirmed multidrug-resistant A. baumannii, with sensitivity only to polymyxin and tigecycline.
- Treatment with high-dose corticosteroids for ES and polymyxin for pneumonia led to significant clinical improvement.
- This case highlights the importance of a multidisciplinary approach in managing complex cases involving rare infections in immunocompromised patients.
- Early identification and appropriate antimicrobial and immunosuppressive management are crucial for favorable outcomes.

The pathophysiology of ES remains unclear; however, autoantibodies suggest an underlying immunological cause^[7]^. Proposed mechanisms include gene mutations in CTLA-4 (CD152) and LRBA proteins, which regulate T-cells. This immune dysregulation is also observed in opportunistic infections. The immune deficiency caused by ES and prolonged immunosuppressant treatment places patients at a high risk of developing respiratory tract infections with an incidence of up to 66.6%^[8]^.

## Case presentation

**Figure 1.**
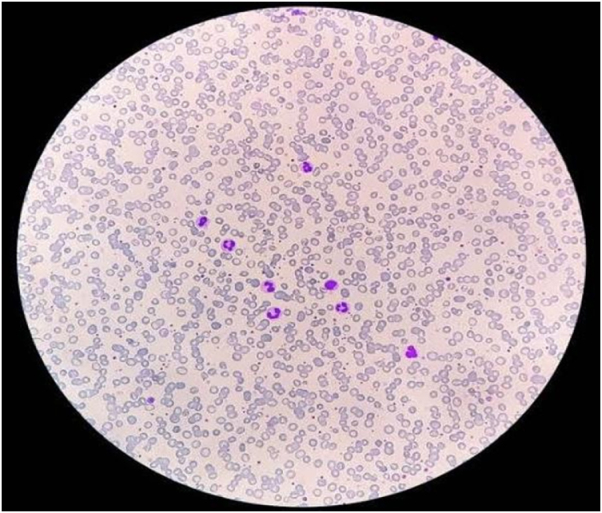
The peripheral smear shows macrocytic red blood cells indicating megaloblastic anemia featuring hyper-segmented neutrophils and a reduced platelet count.

A 19-year-old male with a well-documented history of Evans syndrome, diagnosed 1 year prior, presented to the hospital with acute complaints of fever, shortness of breath, and a productive cough. The patient had been undergoing intermittent immunosuppressive therapy, including corticosteroids and azathioprine, to manage recurrent episodes of autoimmune hemolysis and thrombocytopenia. Over the past month, he reported worsening fatigue, dyspnea, and purulent sputum production, in addition to chest congestion and back pain. Upon physical examination, the patient demonstrated a body temperature of 101°F, tachycardia with a pulse rate of 113 beats per minute, hypotension recorded at 90/70 mmHg, and oxygen saturation of 95%. Clinical assessment revealed significant pallor and slight conjunctival icterus, alongside oral mucosal bleeding, petechiae, and purpura on both upper and lower extremities. Auscultation of the lungs revealed bilateral crackles in the lower lobes. The abdominal examination revealed hepatosplenomegaly. Initial laboratory investigations revealed a hemoglobin level of 7.7 g/dL, with a mean corpuscular volume (MCV) of 118.8 fL and a mean corpuscular hemoglobin (MCH) of 45 pg. Blood smear analysis confirmed the presence of macrocytic red blood cells (Fig. 1). The vitamin B12 level was found to be markedly low at 100 pg/mL, which further supported the presence of macrocytosis. The platelet count was critically low at 4 × 10^9^/L, and the white blood cell count was markedly elevated at 22 × 10^9^/L, with differential analysis showing thrombocytopenia and neutrophilia (Table 1).

**Table 1:** Laboratory investigation of the patient

	Reference value	Week 1	Week 2	Week 3	Week 4	Week 5
Haemoglobin (g/dL)	14–18	7.7	10.2	11.4	11.1	13.2
White blood cells (WBCs) (/L)	4.511 × 10^9^	22 × 10^9^	16.78 × 10^9^	11.6 × 10^9^	7.41 × 10^9^	9.5 × 10^9^
Platelets (/L)	150–400 × 10^9^	4 × 10^9^	12 × 10^9^	16 × 10^9^	27 × 10^9^	44 × 10^9^

Additionally, the erythrocyte sedimentation rate (ESR) and C-reactive protein (CRP) levels were significantly elevated at 50 mm/hr and 26.6 mg/dL, respectively, indicating an active inflammatory process.

**Figure 2.**
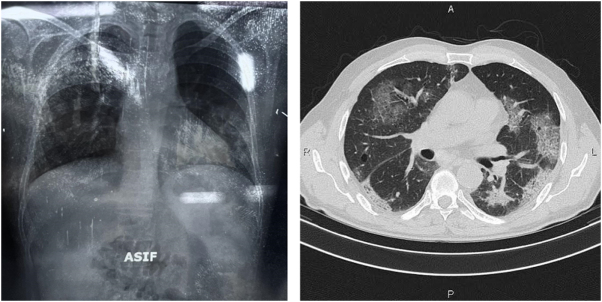
The chest X-ray shows bilateral infiltration.

The coagulation panel demonstrated a prothrombin time of 12.9 seconds. Additional biochemical tests revealed elevated total bilirubin levels (5.73 mg/dL), direct bilirubin (4.1 mg/dL), Alanine aminotransferase (60 U/L), and Alkaline phosphatase (400 U/L). While initial blood cultures were negative, sputum cultures identified *Acinetobacter baumannii*, which exhibited resistance to multiple antibiotics, including amikacin, ciprofloxacin, and ceftriaxone, but remained sensitive to tigecycline and polymyxin. To rule out tuberculosis, GeneXpert and AFB tests were performed, both of which yielded negative results. Radiographic evaluation revealed bilateral infiltrates on chest X-ray, with a computed tomography (CT) scan confirming areas of consolidation (Fig. 2).

The CT scan shows consolidation.

## Diagnosis

The patient has community-acquired pneumonia caused by *Acinetobacter baumannii*, evidenced by fever, productive cough, and consolidation on imaging. He also presents with exacerbated Evans syndrome, characterized by autoimmune hemolytic anemia (hemoglobin of 7.7 g/dL, positive direct Coombs test) and severe thrombocytopenia (platelet count of 4 × 10^9^/L), along with macrocytic anemia secondary to vitamin B12 deficiency.

## Management

Managing Evans syndrome and pneumonia associated with *Acinetobacter baumannii* requires a multidisciplinary approach involving infectious disease specialists, hematologists, and pulmonologists.

### Community-acquired acinebacter pneumonia

The patient was initially managed with vancomycin and meropenem, targeting potential multidrug-resistant pathogens. However, despite this empirical therapy, the patient did not demonstrate any significant clinical response.

Given the lack of improvement and concern for multidrug-resistant *Acinetobacter*, the treatment regimen was revised based on the blood culture report, and we initiated polymyxin agents known for their efficacy against resistant strains of *Acinetobacter*. The patient responded on the fifth day of drug initiation; his fever spikes subsided, and the severity of his cough decreased. Renal function was monitored due to the nephrotoxic risk of polymyxin.

### Evans syndrome

The patient was managed with high-dose methylprednisolone, followed by oral prednisone, to control autoimmune hemolytic anemia and thrombocytopenia. Azathioprine, a steroid-sparing immunosuppressant, was also initiated to maintain remission and reduce long-term corticosteroid dependency. However, due to the onset of pneumonia and subsequent sepsis, azathioprine was discontinued. The patient’s clinical course was closely monitored with regular blood counts and liver function tests to assess the effectiveness of treatment and identify potential adverse effects.

### Macrocytic anemia

To manage the patient’s vitamin B12 deficiency, intramuscular (IM) vitamin B12 injections were initiated. The patient has demonstrated significant improvement in response to this treatment regimen.

## Discussion

We present a rare case of Evans syndrome (ES) complicated by community-acquired pneumonia caused by *Acinetobacter baumannii* in a young adolescent male. ES is an uncommon autoimmune condition defined by the coexistence of autoimmune hemolytic anemia (AIHA) and immune thrombocytopenia (ITP), with or without neutropenia. Its etiology remains unclear, though dysregulated B-cell immunity and autoantibody-mediated destruction of blood cells play central roles^[9]^. ES may occur as a primary idiopathic condition or as a secondary manifestation associated with systemic lupus erythematosus, autoimmune lymphoproliferative syndrome (ALPS), common variable immunodeficiency, or lymphoproliferative malignancies^[10,11]^.

Several studies have demonstrated the chronic, relapsing nature of ES and its association with significant morbidity. Michel *et al*^[10]^, in a cohort of 68 adults, reported frequent relapses, poor response to steroids, and high cumulative morbidity compared to isolated AIHA or ITP. In children, long-term follow-up has revealed frequent relapses, the need for multiple lines of therapy, and mortality in a subset of patients, underscoring the disease’s seriousness^[12]^. More recently, Fattizzo *et al*^[4]^ showed that about one-third of adults with ES experienced severe infections, most commonly bacterial pneumonia, with worse outcomes in secondary ES cases or those requiring multiple immunosuppressants. These findings emphasize that infection is a central feature of the disease trajectory.

First-line therapy for ES usually consists of corticosteroids and/or intravenous immunoglobulin (IVIG), with second-line options such as rituximab, mycophenolate mofetil, cyclosporine, and azathioprine reserved for refractory cases^[13]^. However, immunosuppressive therapies, while often necessary, predispose patients to opportunistic and severe infections. Our patient had been treated with prolonged steroids and azathioprine, likely contributing to his susceptibility. Pan *et al*^[14]^ previously described a case of ES complicated by severe pulmonary tuberculosis, illustrating how opportunistic pathogens exploit the immunocompromised state.

In our case, sputum cultures identified *A. baumannii*, a pathogen typically associated with nosocomial infections but rarely reported as a cause of community-acquired pneumonia (CAP). Although uncommon, community-acquired *A. baumannii* (CA-Ab) pneumonia has been documented, particularly in tropical climates and among immunocompromised hosts^[15,16]^. These infections often present fulminantly, with rapid progression to respiratory failure and high mortality. A case series described fulminant CAP due to *A. baumannii* with several fatalities, noted for its aggressive nature^[16]^. Similarly, Yang *et al*^[17]^ reported necrotizing pneumonia in an immunocompromised patient, emphasizing its destructive potential.

Therapeutic options for *Acinetobacter* infections are limited by multidrug resistance. Standard treatments include carbapenems, aminoglycosides, and beta-lactamase inhibitor combinations; however, resistance has increasingly necessitated the use of polymyxins^[18]^. In our patient, empiric vancomycin and meropenem failed to improve symptoms, and escalation to polymyxin resulted in a clinical response, although with the risk of nephrotoxicity, which required vigilant monitoring^[19]^. Iwasawa *et al*^[20]^ demonstrated the importance of early empiric recognition by successfully treating CA-Ab pneumonia with meropenem based on Gram-stain findings, emphasizing that early suspicion can be lifesaving.

The coexistence of ES and CA-Ab pneumonia creates a dual risk scenario. On one hand, ES predisposes to infection through immune dysregulation and treatment-induced immunosuppression; on the other hand, infection itself may trigger relapse of cytopenias, perpetuating a vicious cycle. While we found no prior reports of ES complicated specifically by *A. baumannii*, Sawant *et al*^[21]^ described pneumonia unmasking ES, and similar infection-driven exacerbations have been reported in tuberculosis^[14]^. This case highlights three important clinical lessons. First, physicians should maintain a high index of suspicion for atypical pathogens in immunocompromised ES patients presenting with pneumonia. Second, rapid microbiological testing and early adjustment of therapy to target resistant organisms are essential. Third, immunosuppressive therapy must be carefully balanced: controlling cytopenias is vital, but clinicians must remain vigilant of the increased infection risk.

In summary, this case highlights the vulnerability of ES patients to severe and unusual infections such as community-acquired *A. baumannii* pneumonia. It reinforces the need for comprehensive infection risk assessment, judicious use of immunosuppression, early microbiological diagnosis, and timely escalation of antimicrobial therapy. Prospective registries are needed to clarify infection patterns, outcomes, and optimal practices for managing this challenging overlap of autoimmune cytopenia and opportunistic infection.

## Conclusion

This case report highlights the complexities associated with Evan’s syndrome, particularly due to the given immunocompromised state, which makes it easier for opportunistic organisms like *Acinetobacter baumannii* to cause further complications. It is important that vigilant monitoring and tailored therapeutic approaches are implemented to prevent further complications. Further research is warranted to better understand the disease mechanism and optimum treatment strategies to improve disease outcomes.

## Limitations and delimitations

The limitations of this case report include the generalizability of the findings, as the results are based on a single patient with a rare combination of community-acquired *Acinetobacter* pneumonia and Evans syndrome, which may not reflect the broader population. The diagnostic challenges faced, including delayed identification of the infection due to its multidrug-resistant nature, further complicate treatment protocols. Additionally, the effectiveness of polymyxin therapy, though beneficial in this case, may not be applicable to all patients due to potential nephrotoxicity. The patient’s response to treatment may also vary compared to others with similar conditions, limiting the predictability of outcomes. The delimitations include the focus on this singular case, which restricts the scope to this specific instance and does not address other potential variations of the disease or treatment outcomes. The report is also limited to the clinical course of one patient, a 19-year-old male, which may not encompass the experiences of other demographic groups. Lastly, the treatment protocols discussed are specific to this case and may not be broadly applicable, while the timeframe covered does not consider long-term outcomes beyond the period of observation.

## Data Availability

Not applicable.
